# Structure-Based Virtual Screening, Molecular Dynamics and Binding Free Energy Calculations of Hit Candidates as ALK-5 Inhibitors

**DOI:** 10.3390/molecules25020264

**Published:** 2020-01-09

**Authors:** Sheila C. Araujo, Vinicius G. Maltarollo, Michell O. Almeida, Leonardo L. G. Ferreira, Adriano D. Andricopulo, Kathia M. Honorio

**Affiliations:** 1 CCNH, Federal University of ABC, Santo Andre, SP 09210-580, Brazil; sheila.shalala@gmail.com; 2 Department of Pharmaceutical Products, Faculty of Pharmacy, Federal University of Minas Gerais, Belo Horizonte, MG 31270-901, Brazil; viniciusmaltarollo@gmail.com; 3 IQSC-USP, Sao Carlos, SP 13563-120, Brazil; michelloliveira02@gmail.com; 4 Laboratory of Medicinal and Computational Chemistry, Physics Institute of Sao Carlos, University of Sao Paulo, Sao Carlos, SP 13563-120, Brazil; leonardo@ifsc.usp.br (L.L.G.F.); aandrico@ifsc.usp.br (A.D.A.); 5 EACH, University of São Paulo, Sao Paulo, SP 03828-000, Brazil

**Keywords:** cancer, ALK-5, SBVS, molecular dynamics, binding free energy calculations

## Abstract

Activin-like kinase 5 (ALK-5) is involved in the physiopathology of several conditions, such as pancreatic carcinoma, cervical cancer and liver hepatoma. Cellular events that are landmarks of tumorigenesis, such as loss of cell polarity and acquisition of motile properties and mesenchymal phenotype, are associated to deregulated ALK-5 signaling. ALK-5 inhibitors, such as SB505154, GW6604, SD208, and LY2157299, have recently been reported to inhibit ALK-5 autophosphorylation and induce the transcription of matrix genes. Due to their ability to impair cell migration, invasion and metastasis, ALK-5 inhibitors have been explored as worthwhile hits as anticancer agents. This work reports the development of a structure-based virtual screening (SBVS) protocol aimed to prospect promising hits for further studies as novel ALK-5 inhibitors. From a lead-like subset of purchasable compounds, five molecules were identified as putative ALK-5 inhibitors. In addition, molecular dynamics and binding free energy calculations combined with pharmacokinetics and toxicity profiling demonstrated the suitability of these compounds to be further investigated as novel ALK-5 inhibitors.

## 1. Introduction

Cancer is associated to genetic mutations that provide specific characteristics to the affected cells, such as high levels of proliferation, metastasis, and evasion of apoptosis. Activin-like kinase 5 (ALK-5), also known as TGF-beta type I receptor (TGFβRI), is a transmembrane receptor involved in the development of various types of malignancies, such as pancreatic carcinoma, cervical cancer, and liver hepatoma [1,2,3,4,5]. TGFβ is a multipurpose cytokine that affects biological processes as regulation of cell proliferation and differentiation, immune response, angiogenesis and apoptosis. Therefore, deregulation of TGFβ dependent pathways leads to life-threatening diseases such as cancer [1,2,3,4,5,6,7,8,9].

The complex function of TGFβ depends on the activation of two highly conserved single transmembrane serine/threonine kinases: type I (TGFβRI or ALK-5) and type II (TGFβRII) receptors. The mechanism of TGF-β activation involves the following steps: TGFβRII phosphorylates threonine residues in the GS (repeated series of glycine-serine) domain of ligand-occupied ALK-5 that occurs in the portion localized outside the membrane [10,11]. Then, ALK-5 phosphorylates the cytoplasmic proteins SMAD2 (small mothers against decapentaplegic type 2) and SMAD3 (small mothers against decapentaplegic type 3) at two carboxyl terminals of serine residues. The phosphorylated SMAD proteins form heteromeric complexes with SMAD4; this complex moves into the nucleus to determine gene transcription [12,13,14,15]. Inhibitors targeting ALK-5, such as SB505154 [16], GW6604 [17], SD208 [18], and LY2157299 [19], act by inhibiting the autophosphorylation of ALK-5 and inducing the transcription of matrix genes [20]. Moreover, ALK-5 inhibitors have improved clinical changes consistent with a reduction of epithelial–mesenchymal transition (EMT), which is one of the landmarks of tumorigenesis. Despite the promising results that have been recently reported, no ALK-5 inhibitor has reached the market yet [21,22].

Currently, virtual screening is a worthwhile approach to the discovery of new hits. Structure-based virtual screening (SBVS) is a cost-effective tool to identify and prioritize ligands for subsequent in vitro and in vivo profiling, and, therefore, is an attractive way to prospect novel ALK-5 inhibitors [4,13,14,23,24]. Here, a SBVS workflow was designed to probe a large chemical library from the ZINC database [25]. The first stage of the developed SBVS protocol involved the selection of compounds according to the main intermolecular interactions at the binding site (in particular, interactions with His-283 and Lys-232). After several analyses on the essential ligand-receptor interactions, five compounds were selected for further prediction of their absorption, distribution, metabolism, excretion and toxicity (ADME-Tox) profiles. In this step, a known ALK-5 inhibitor (4-([1,2,4]triazolo [1,5-a]pyridin-6-yl)-*N*-(4-methoxyphenyl)-3-(6-methylpyridin-2-yl)-1*H*-pyrazole-1-carbothioamide) was used as a reference (benchmark) for the pharmacokinetics and toxicity studies [26]. Next, molecular dynamics simulations and binding free energy calculations were performed to evaluate the stability and energetics associated to the formation of the receptor-ligand complexes.

## 2. Methodology

### 2.1. Molecular Docking

Thirteen X-ray structures (Table S1) of ALK-5 deposited in the Protein Data Bank (PDB) were analyzed using some parameters in order to select the most suitable ALK-5 structure for our SBVS study, such as information on the accuracy of crystallographic structures (R-factor and R-free) [27,28,29], as well the similarity values and physicochemical properties of all ligands (Tables S1–S3).

USCF Chimera 1.6.1 [30] was used to prepare the target structures and docking simulations were performed using DOCK 3.5.4 [31] with the aim of predicting the way in which the ligands bind to ALK-5. It is important to mention that some studies in literature have assessed the success rate of docking programs by applying redocking tests, whereby a ligand is docked into the protein structure from which it was extracted [27,28]. Other important validation test is cross-docking (process in which the crystallographic ligand is simulated in other crystal structures of the same biological target), since it provides a more realistic assessment of the docking program’s ability to reproduce X-ray results and information on effects related to induced fit [27,28]. The later procedure can also be considered as a consensus analysis. These two approaches were used to validate our SBVS workflow. Hence, the crystallographic ligand at the PDB structure (1RW8) was used in the redocking simulations. For the cross-docking tests, we used the ligands 855 (3HMM) and P1Y (1PY5), which were docked in the structure 1RW8. The preparation of the receptor structure consisted of adding hydrogen atoms and setting the protonation state of some residues. All water molecules were removed, since there are no structural water molecules mediating receptor–inhibitor interactions.

Shortly, we selected the ALK-5 structure with PDB ID 1RW8 [24] (resolution: 2.4 Å) due to its X-ray quality and the similarity of its crystallographic ligand to a previously studied compound set [32]. The 3D structures of the ligands used for this validation were selected from ZINC as flexibase files (the file type containing the conformer library of compounds, which is recognized by DOCK 3.5.4). The compound poses were classified according to the docking algorithms’ ability to find the compound pose with the lowest force field values, including scoring function based on van der Waals and electrostatic components [33,34].

The binding site considered in the docking simulations was defined based on the coordinates of the crystallographic ligand (sphere centered in 8 Å from the crystal ligand). Finally, docking simulations considering the receptor structure as rigid and multiple poses of the compound library were performed by using DOCK 3.5.4. [30,31,32,33,34,35,36]. Each ligand-receptor complex was classified according to ChemGrid function that produces force field scores or mechanics interaction energies calculated as a sum of van der Waals (using a modified AMBER potential function) and electrostatic interaction energies (using potentials calculated with DelPhi) corrected for the ligand desolvation [33,37]. It is important to note that the definition of the binding site and the flexibility degree of ligands (bonds and angles) can also affect the molecular docking success rate because these factors change the search space covered by the docking algorithm [38,39,40,41].

### 2.2. Structure-Based Virtual Screening

**Figure 1:**
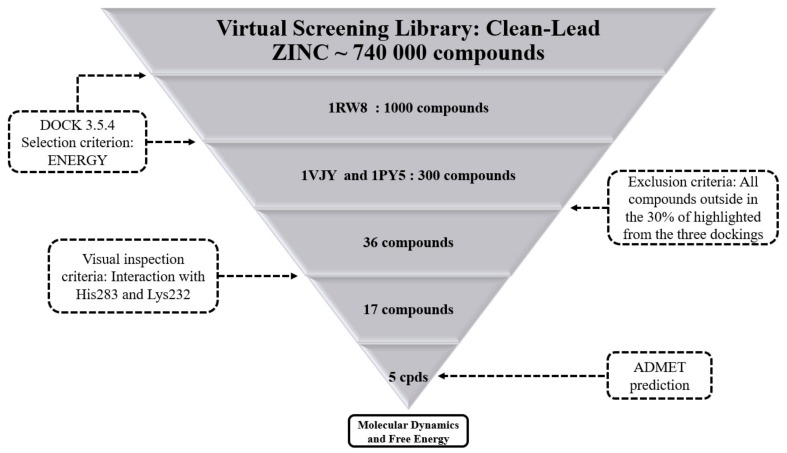
Schematic representation of the SBVS strategy employed in this study. The selection of 1000 compounds were performed from the force field scores and submitted to other docking analyses. Subsequently, 300 compounds were selected according to the scores from three docking simulations applied with the following PDB structures: 1RW8, 1PY5, and 1VJY. Subsequently, the 30% top scored compounds simultaneously obtained from all three docking simulations (at the PDB structures 1RW8, 1PY5 and 1VJY) were selected. Then, we carried out a visual inspection regarding the main molecular interactions between some important amino acid residues (His283 and Lys232) at the binding site of ALK-5 and the studied ligands. Afterwards, we performed MD simulations and binding free energy calculations at the ATP binding site of ALK-5.

Figure 1 presents the SBVS workflow employed to find hits as ALK-5 inhibitors. The clean-lead subset (740,000 compounds) containing a large compound library, available in ZINC (version of December-2011) [25], was chosen for the SBVS studies. This subset includes purchasable compounds with molecular weights between 250 and 350 Da, logarithms of n-octanol/water partition coefficient between 2.5 and 3.5, and the number of rotatable bonds between 5 and 7; this subset does not present toxic compounds (aldehyde and thiol groups) [25].

From the selected ALK-5 structure (1RW8), the molecular docking simulations were performed using ZINC clean-leads subset and docking runs using DOCK 3.5.4, and the 1000 top-scored molecules with the lowest force field scores were selected for the next stage of the SBVS protocol. Then, these 1000 hit compounds were submitted to additional docking runs using other two ALK-5 structures (1PY5 and 1VJY) to evaluate the influence of possible conformational differences in the score ranking of the compounds. After that, 300 hit compounds were selected based on their force field scores against ALK-5 structures (1RW8, 1PY5, and 1VJY). Subsequently, a consensus analysis was performed and the 30% top scored compounds simultaneously obtained from all three docking simulations were selected. Next, thirty-six compounds (approximately 0.005% of the initial set) were selected by visually inspecting the docking results, focusing on the main interactions between some essential residues at the binding site of ALK-5 and the selected compounds. For the visual inspection, the presence of polar interactions with His283 and Lys232 was used as a selection criterion, in addition to other polar interactions and stereochemical complementarity. Finally, we performed the prediction of pharmacokinetics and toxicity data for the five hit compounds and compared to a known benchmark ALK-5 ligand (4-([1,2,4]triazolo[1,5-a]pyridin-6-yl)-*N*-(4-methoxyphenyl)-3-(6-methylpyridin-2-yl)-1*H*-pyrazole-1-carbothioamide) [21,31,33,35,36]

### 2.3. Prediction of Pharmacokinetic (PK) Parameters

ADME-Tox (Absorption, Distribution, Metabolism, Excretion, and Toxicity) studies are very important in drug discovery and are used to assist the optimization of lead compounds [37]. Although in silico ADME-Tox descriptors are not as accurate as in vivo or in vitro experiments, they provide useful information to assess the drug-likeness of compounds. In this study, to evaluate the drug-likeness of the selected hits, physicochemical, molecular, pharmacokinetic (PK) and toxicity parameters were predicted by using pkCSM [38]. This prediction tool provides a rapid and simple way to evaluate the pharmacokinetic profile of hits. Thus, we compared the PK properties of the molecules selected from SBVS with the PK parameters of the crystallographic ligand [38].

### 2.4. Molecular Dynamics (MD) Simulations

Five ALK-5-ligand complexes selected from the SBVS studies were subjected to molecular dynamics simulations, employing Amber18 [39]. In this way, the five systems were prepared, and the first step involved the calculation of ligand’s charges (RESP: Restrained electrostatic potential) [40]. The RESP charges of the five ligands were calculated by using HF/6-31* [41] in Gaussian09 [42]. The next step related to the MD simulations was the preparation of the complexes in the Tleap module (implemented at Amber18) [39]. The solvent box used in this study was the octahedral with transferable intermolecular potential with three points (TIP3P waters), the distance between the walls of the solvent box and the biological target (ALK-5) was 12 Å and the force field 99SB was employed with Particle Mesh Ewald Molecular Dynamics (PMEMD) code [43].

After the preparation of the protein-ligand complexes, the minimization of the structures was performed. Next, the ligand-receptor complexes were heated (0–300 K, 50 ps) and submitted to equilibration (10 ns). The final step of the MD simulation is related to the production process, which was operated along 100 ns, with the isothermal-isobaric ensemble, pressure and temperature (NPT) conserved, Langevin Thermostat (ntt = 3), barostat of Monte Carlo, and 1 atm pressure. After 100 ns of simulation, the results were analyzed in terms of the root mean square deviation (RMSD) and root mean square fluctuation (RMSF). Finally, the binding free energies of the five complexes were calculated to assess the most promising ALK-5 inhibitors.

### 2.5. Calculations of Binding Free Energy

Using the ligand-receptor conformations obtained from the MD simulations, the binding free energy was calculated for the five systems, in which the snapshots from 0 to 250 of the most stable trajectories (analyzed from the RMSD values) were used. Thus, the technique known as SIE (solvated interaction energy), which is implemented in the SIETRAJ program, was used for the calculation of ΔG [44]. Equation (1) presents the parameters that the SIE method uses for the calculation of ΔG:

(1) $$\Delta G_{\mathrm{bind}}=\alpha [E_{inter}^{Coul}(D_{in})+\Delta G_{desolv}^{R}(D_{in})+E_{inter}^{vdW}+\gamma (\rho ,\;D_{in})\Delta \mathrm{MSA}\;(\rho )]+C$$

The terms in Equation (1) can be defined as: Coulomb interaction energy ($E_{inter}^{Coul}$); free energy of desolvation ($\Delta G_{desolv}^{R}$); non polar free energy of desolvation ($E_{inter}^{vdW}$); derivation factor of the atomic radius of Born (ρ); solute pool dielectric constant (D*_in_*); coefficient of surface tension (γ) and the molecular surface area of solute upon binding (ΔMSA) [44,45,46]. Using the SIE method, the calculated ∆G values were used to indicate the stability of the five ligands at the binding site of ALK-5.

## 3. Results and Discussion

### 3.1. Molecular Docking

As previously described, the analysis of several crystallographic structures (Table S1) available at PDB was essential to assist the selection of the most suitable ALK-5 structure for our virtual screening. Furthermore, the knowledge about the features of the binding site (for example, the main amino acid residues as Lys232 and His383) and other polar interactions were crucial for this selection.

**Figure 2:**
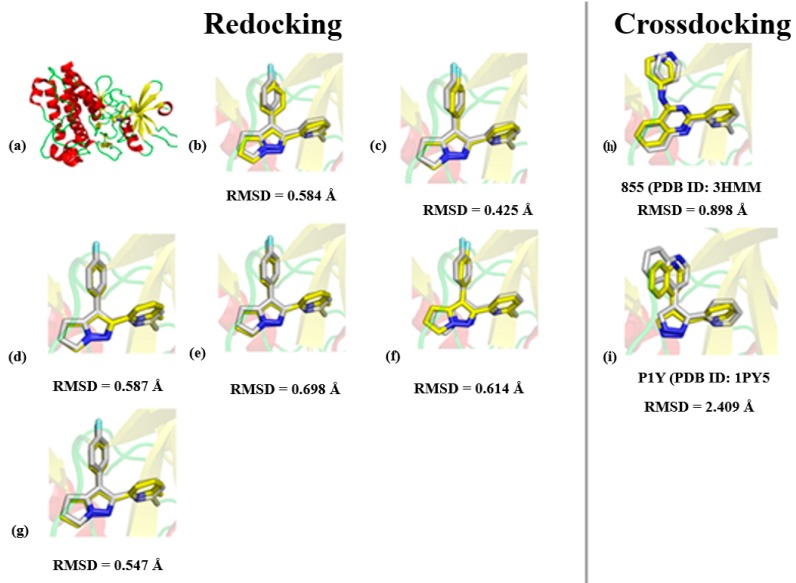
(**a**) ALK-5 structure used in the docking simulations (1RW8). The different poses for all crystallographic ligands are described according to the RMSD values calculated from USCF Chimera 1.6.1; (**b**–**g**) redocking results taking into account the best poses of the crystallographic ligand at 1RW8; and (**h**,**i**) cross-docking results using crystallographic inhibitors from different PDB structures of ALK-5. The crystallographic ligand is displayed in white and the docking poses in yellow.

From the redocking and cross-docking results displayed in Figure 2, we observe that the algorithm and, consequently, all parameters successfully reproduced the crystallographic conformation of the ligand and the main interactions at the binding site of ALK-5. The cross-docking experiments also showed the ability of the docking protocol to predict the most suitable binding mode. Although the RMSD values can be considered relatively high, the cross-docking experiments with P1Y correctly predicted the position of polar groups and rings at the binding pocket (Figure 2i). Thus, the redocking and cross-docking experiments validated the algorithm used for our SBVS study. The next step involved several SBVS runs to prospect new ALK-5 inhibitors. The poses of the hit compounds obtained during the SBVS protocol were ranked according to the docking algorithms´ ability to find the compound pose with the lowest force field values, including scoring functions based on van der Waals and electrostatic components [33,47].

### 3.2. Structure-Based Virtual Screening and Binding Analysis of the Selected Hits

**Figure 3:**
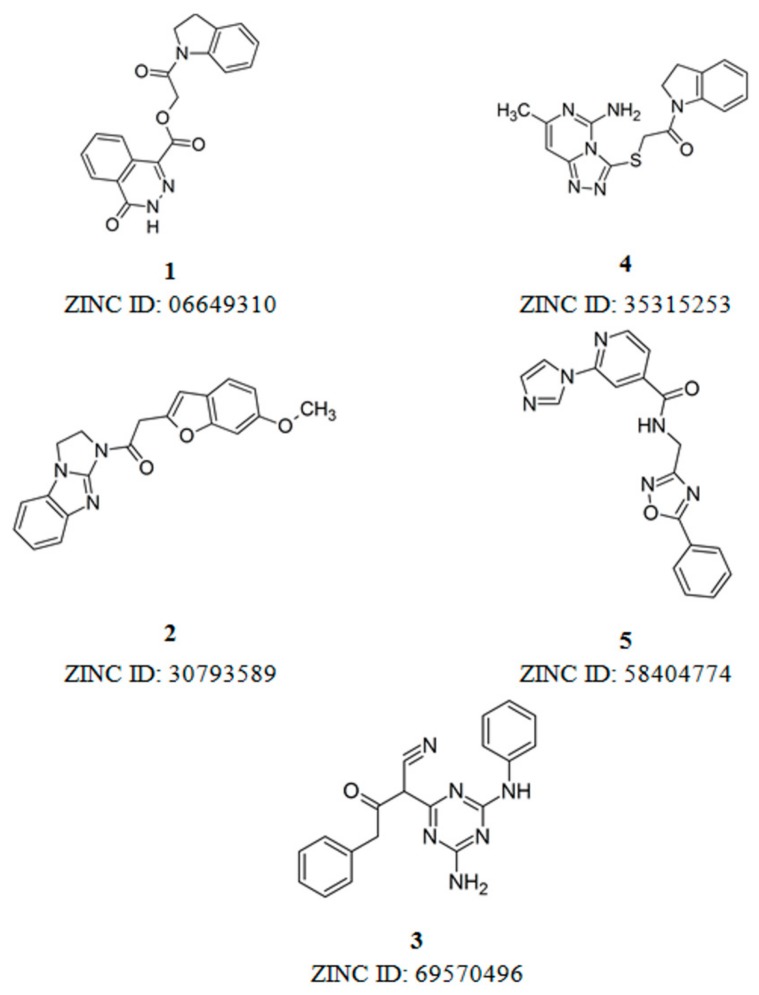
Compounds selected from SBVS.

**Figure 4:**
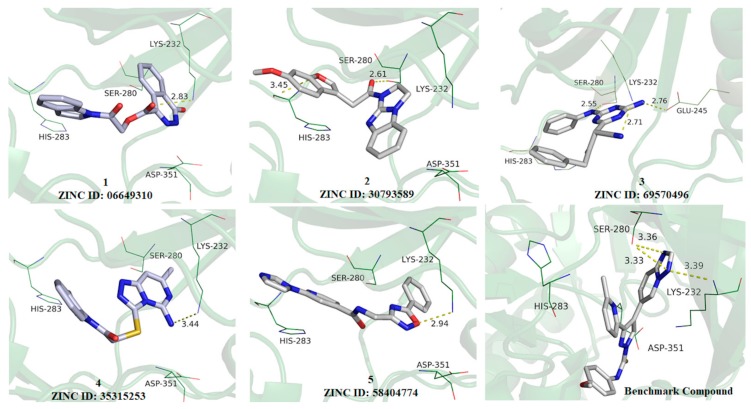
Molecular docking analysis for the five hits (**1**–**5**) selected from SBVS and the benchmark compound. The hydrogen bond (H-bond) interactions between the five hits and the main amino acid residues are shown in yellow dots (distance in angstroms).

After all phases described in Figure 1, five hits were selected from our SBVS protocol and their structures are displayed in Figure 3. In addition, the five hits selected were examined in terms of its binding mode and intermolecular interactions with important amino acid residues at the binding site of ALK-5 (Figure 4).

All five compounds at the binding site of ALK-5 are shown in Figure 4. The main difference between the five hits is the number of H-bond donor and acceptor groups, for example, the amide group; there are at least two H-bond donor groups. We can see an important polar interaction between the ester group of the ligand 1 and the terminal amine of Lys232, which is an essential residue involved in ALK-5-ligand polar contacts. Analyzing ligand 2, the oxygen atom of benzofuran moiety (Figure 4) interacts with His283 as well as there is an interaction between the ketone oxygen and Ser280. These two amino acid residues are known to be essential for the formation of hydrogen bonds with the hinge region. Ligand 3 (Figure 4) establishes polar interactions between its nitrile nitrogen and Lys232; the triazine ring of the compound **3** interacts with Glu245 and the amine group performs a polar interaction with Ser280. In addition to Lys232, Glu245, and Ser280, as the main residues of the ALK-5 active site, the presence of amine and amide groups in the ligands can increase the number of hydrogen bonds and, probably, improve the biological response. Thus, the ADME-Tox profile of the five selected molecules was also predicted (Supplementary Material) and compared to a known (benchmark) ALK-5 ligand (4-([1,2,4]triazolo[1,5-a]pyridin-6-yl)-N-(4-methoxyphenyl)-3-(6-methylpyridin-2-yl)-1H-pyrazole-1-carbothioamide) [21,35,36,48].

### 3.3. ADME-Tox Profile

For the five compounds screened, the compound **1** was predicted to have good human intestinal absorption (HIA = 99.017% absorbed), with Caco-2 permeability of 1.229 (log Papp in 10^−6^ cm/s; Papp is the apparent intrinsic permeability) and water solubility (expressed as the logarithm of the molar concentration) equals to −4.206 log mol/L [49]. Similarly, for the benchmark compound, HIA, Caco-2 permeability and water solubility were equal to 100% absorbed, 1.145 (log Papp in 10^−6^ cm/s) and −2.873 log mol/L, respectively. The property “Distribution” was considered in terms of four predictors: VDss (volume of distribution at steady state), fraction unbound, blood-brain-barrier (BBB), and central nervous system (CNS) permeability. VDss represents the range of drug distribution in tissues. Unbound fraction gives the concentration of drug that is not bound to plasma proteins and, then, is available to exert the pharmacological effect. BBB permeability is the ability of a compound to access the CNS. The ADME-Tox predictions indicated that compound **1** has good distribution properties, which is a desirable feature for a safe and effective drug candidate. Moreover, efficacy and toxicity are affected by drug metabolism. Diverse cytochrome P450 (CYP) enzymes are involved in drug metabolism and pkCSM predicts sites of metabolism considering various CYP isoforms, such as cytochrome P450 2D6 (CYP2D6), cytochrome P450 3A4 (CYP3A4), cytochrome P450 1A2 (CYP1A2), cytochrome P450 2C19 (CYP2C19), and cytochrome P450 2C9 (CYP2C9). Drug candidates are required to be effectively eliminated from the body, resulting in a safe clearance profile, after the therapeutic response is ensured. The ADME-Tox analyses showed that among the five selected hits, compounds **1** and **5** have good clearance. For a more detailed analysis of the behavior of these molecules at the binding site of ALK-5, including their stability and binding energy, MD simulations and binding free energy calculations were performed.

### 3.4. MD Simulations and Analysis of Binding Mode

**Figure 5:**
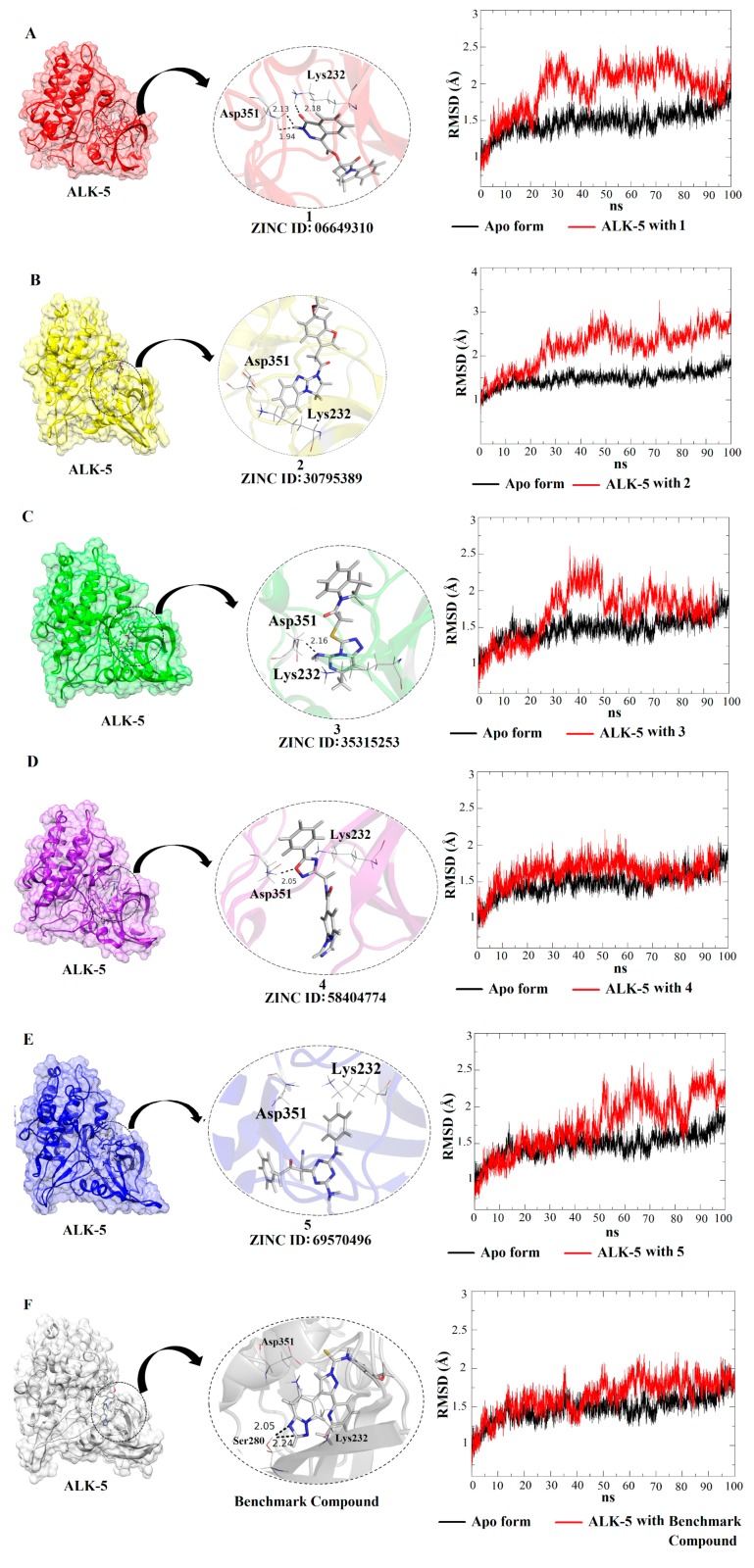
RMSD plots and intermolecular interactions for the five ligand-receptor complexes (**A**–**E**) and the benchmark compound from the structure with PDB ID 1RW8 (**F**).

The five ALK-5-ligand complexes generated from the molecular docking studies (employing the ALK-5 structure with PDB ID 1RW8) were used as the initial conformations for the MD simulations. These MD studies allowed the analysis of the dynamic behavior of the ALK-5-inhibitor complexes including ligand-induced conformational changes in the structure of the enzyme. Six MD simulations were performed: one for each ALK-5-inhibitor complex and one for the apo form of ALK-5. MD simulations of 100 ns were conducted, and the resulting RMSD values were used to evaluate the ligand-induced conformational changes in the structure of ALK-5. The results obtained are in agreement with those described in the literature [50,51,52]. A clustering analysis was also performed using Chimera 1.6.1 [30], which showed the conformation that most often appeared during the MD simulations (100 ns). In addition to the RMSD values, the intermolecular interactions between the five selected ligands and ALK-5 were analyzed using the conformation selected from the clustering analysis. These results and the intermolecular interactions between the five ligands and Lys232 and Asp351 are presented in Figure 5.

**Figure 6:**
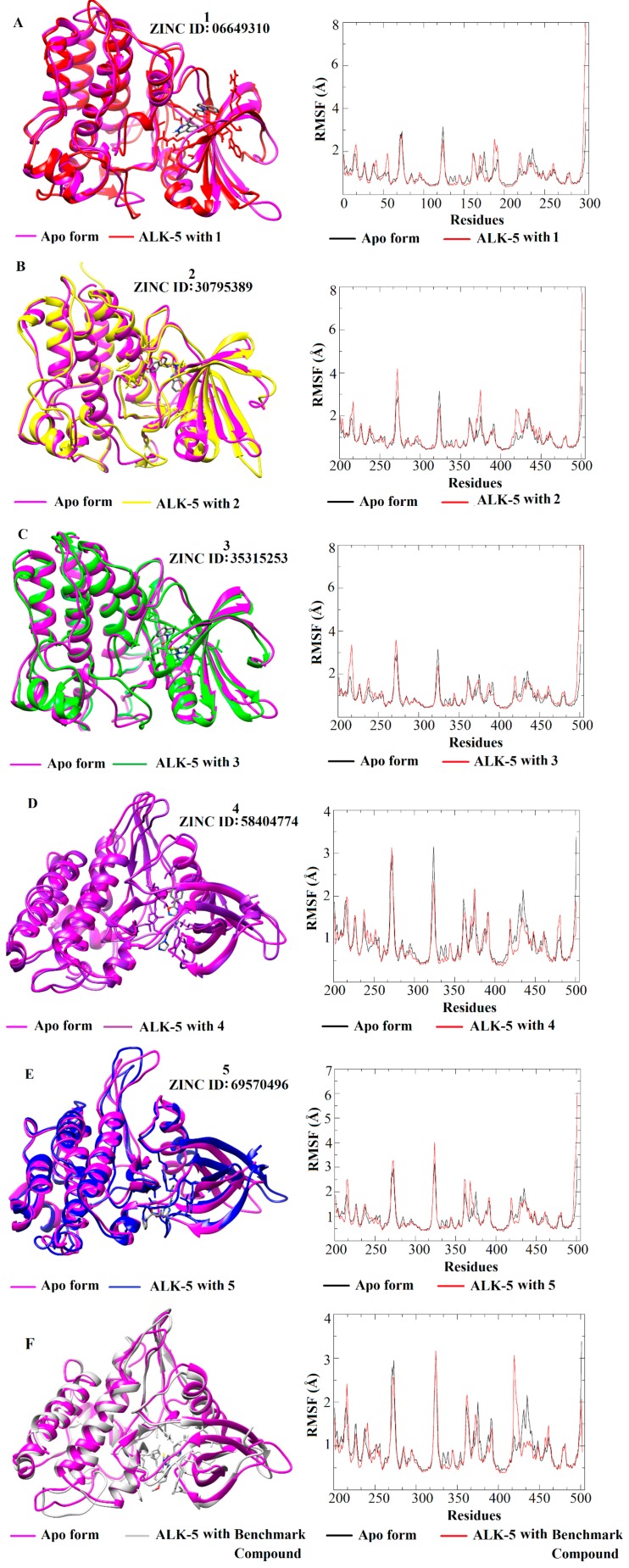
RMSF plots for the five selected ALK-5-ligand complexes (**A**–**E**) and the benchmark compound from the structure with PDB ID 1RW8 (**F**).

Figure 5 shows that all inhibitors induce conformational changes in the structure of ALK-5; the RMSD values demonstrate a lower mobility of the apo form compared to that of the ligand-receptor complexes. The RMSD plots suggest that the complex containing the compound **4** (Figure 5D) presented less mobility since its RMSD values are similar to the apo form; the complexes formed by the compounds **1**, **2**, **3**, and **5** involve more significant conformational changes in the target structure as well as the number of hydrogen bonds. In particular, taking into account the hydrogen bonds, the compound **1** (06649310) makes a higher number of interactions suggesting that this inhibitor can contribute significantly in the ALK-5 inhibition. In addition to the RMSD analysis, RMSF plots were generated for the five receptor-inhibitor complexes and the apo form (Figure 6). These plots indicate that the fluctuations of the residues are larger for the ALK-5-inhibitor 1 complex compared to the apo form. The largest fluctuation is related to the last residue of a loop region. Some small fluctuations are related to key residues such as Lys232 and Asp351. The fluctuations are smaller when the receptor is bound to the compound **4** (Figure 6D), which corroborates the RMSD analysis that shows minor conformational changes upon interaction with this ligand. The RMSF plot for the compound **4** was compared with the apo form of ALK-5.

The RMSD and RMSF plots allowed for the evaluation of the dynamic behavior of the five selected compounds in complex with ALK-5 and the fluctuations of the amino acid residues upon ligand binding. Moreover, these analyses showed that ALK-5 reaches conformational stability after interaction with the selected hits. In fact, the RMSD and RMSF plots show the most significant conformational changes for this receptor structure and the largest number of hydrogen bonds involving key amino acid residues.

### 3.5. Binding Free Energy

After the MD simulations, the most stable sub-trajectories of the five ALK-5-inhibitor structures were selected (20 ns for each complex) for binding free energy calculations using the SIE method. These results are presented in Table 1 along with the ΔG value for the benchmark compound (4-([1,2,4]triazolo[1,5-a]pyridin-6-yl)-*N*-(4-methoxyphenyl)-3-(6-methylpyridin-2-yl)-1*H*-pyrazole-1-carbothioamide), which is a known ALK-5 inhibitor (IC_50_ = 0.57 nM) [53].

The binding free energies were calculated and compared with the values obtained from the docking simulations, as reported in Table 1. From the SIE and the ChemGrid values, we can see that the complexes formed between ALK-5 and the compounds **1** and **2** present the lowest force field score and, therefore, could form the most stable complexes with ALK-5 when compared to the values for the benchmark compound. MD results have also indicated this behavior for these complexes. It is interesting to mention that studies in the literature have shown that the thioamide group is a key player for the inhibition of ALK-5 [1,12] and, the compounds **1** and **2** have amide groups in their structures, which are stronger hydrogen-bond acceptors than thioamide. In addition, the SIE analyses indicated that the other molecules also had promising binding free energy, as well as ChemGrid scores when compared to the benchmark compound. Therefore, the gathered results demonstrate that the selected hits could be considered as putative ALK-5 inhibitors and promising starting points for the design of novel anticancer compounds.

## 4. Conclusions

ALK5 is involved in various pathological processes, such as pancreatic carcinoma, cervical cancer, and liver hepatoma. Following these findings, we designed a SBVS workflow aiming to identify potential ALK-5 inhibitors suitable for further experimental studies. After the first steps involved in SBVS studies, a consensus analysis from the molecular docking results was conducted to select a set of five compounds for further studies. From the selected hits, the compounds **1** and **2** presented interesting docking results that show important hydrogen bonds between these molecules and key residues at the binding site, such as Lys232 and His283. In addition, MD simulations indicate expressive conformational changes of the ALK-5 structure in the presence of compounds **1** and **2**. From the calculation of the binding free energy, the complexes formed by ALK-5 and compounds **1** and **2** had the lowest value of ΔG and were similar to the benchmark compound. The other compounds (**3**–**5**) also deserve attention due to the interaction involving the hinge region of the target and low ΔG values that can finger out to the design of new lead anticancer compounds. It is worth mentioning that ADME-Tox predictions were also performed to evaluate the safety profiles of the selected compounds. Therefore, this study provides a structural and energetic analysis of possible ligands of ALK-5, in particular the compounds **1** and **2**, which are promising hits as structurally novel ALK-5 inhibitors.

## Figures and Tables

**Table 1:** ΔG values and ChemGrid score for the five hits and the benchmark compound.

ALK-5-Ligand	SIE (kcal/mol)	ChemGrid Score (kcal/mol)
1	–9.49	–50
2	–8.24	–51
3	–7.78	–49
4	–8.00	–49
5	–7.50	–45
Benchmark compound	–9.60	–52

## Supplementary Materials

The following are available online, Table S1. 3D structures of ALK-5 available at PDB, their main structural parameters (Resolution, R-value and R-free) and crystal ligands.; Table S2. Similarity values of each selected compound based on the crystal ligand.; Table S3. Physicochemical properties of some ALK-5 ligands* and each crystallographic ligand.; Table S4. Compounds selected from visual inspection regarding the main molecular interactions between some residues in the binding site and the studied ligands.; Table S5. Prediction of absorption (A), distribution (D), metabolism (M) and excretion (E) for the selected compounds and the crystal ligand.; Table S6. Prediction of toxicity for the new proposed compounds and the crystal ligand.
