# Supplementary material for: Structure-Based Virtual Screening, Molecular Dynamics and Binding Free Energy Calculations of Hit Candidates as ALK-5 Inhibitors
Source: Molecules. 2020 Jan 9;25(2):264. doi: 10.3390/molecules25020264 (PMC7024315; doi:10.3390/molecules25020264)
Supplement: Supplementary file 1 [file molecules-25-00264-s001.pdf]

Supplementary Material

Table S1. 3D structures of ALK-5 available at PDB, their main structural parameters (Resolution, R-value and R-free) and crystal ligands

	Resolution (Å)	R-value	R-Free	Ligand	
3HMM	1.70	0.223	0.241	855: 2-(6-methylpyridin-2-yl)-N-pyridin-4-ylquinazolin- 4-amine	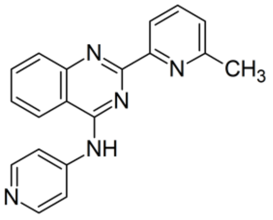
3GXL	1.80	0.228	0.262	QIG: N-1H-indazol-5-yl-2-(6-methylpyridin-2-yl)quinazolin- 4-amine	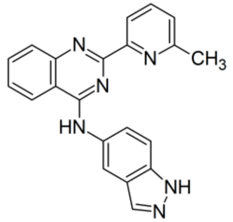
2WOT	1.85	0.181	0.221	ZZG: 4-[(5,6-dimethyl-2,2'-bipyridin-3-yl)oxy]-n-(3,4,5-trimethoxyphenyl)pyridin-2-amine	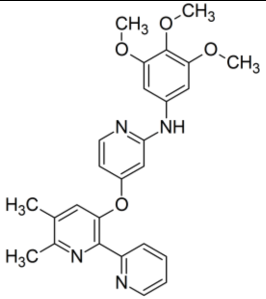

1VJY	2.00	0.223	0.275	460: 2-[5-(6-methylpyridin-2-yl)-2,3-dihydro-1h-pyrazol-4-yl]-1,5-naphthyridine	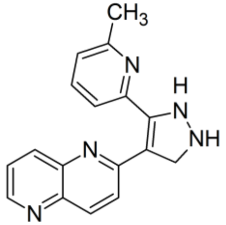
2WOU	2.30	0.229	0.302	ZZF: 4-({4-[(2,6-dimethylpyridin-3-yl)oxy]pyridin- 2-yl}amino)benzenesulfonamide	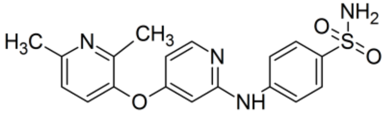
1PY5	2.30	0.258	0.292	PY1: 4-(3-pyridin-2-yl-1h-pyrazol-4-yl)quinoline	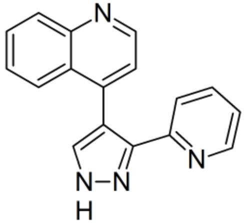
1RW8	2.40	0.260	0.291	580: 3-(4-fluorophenyl)-2-(6-methylpyridin-2-yl)- 5,6-dihydro-4h-pyrrolo[1,2-b]pyrazole	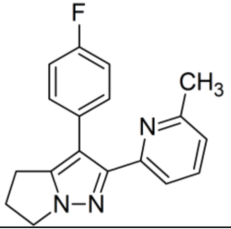
3KCF	2.80	0.237	0.278	JZO: 4-[3-(methoxymethyl)phenyl]-1,2-dimethyl- 5-quinoxalin-6-yl-1,2-dihydro-3H-pyrazol- 3-one	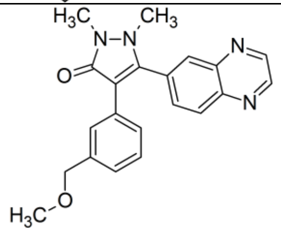

3FAA	3.35	0.228	0.271	55F: N-[4-(5-fluoro-6-methylpyridin-2-yl)-5-quinoxalin- 6-yl-1H-imidazol-2-yl]acetamide	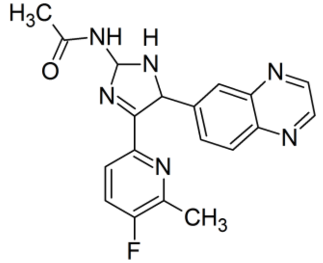
2X7O[68]	3.70	0.267	0.273	ZOP: (3z)-n-ethyl-n-methyl-2-oxo-3-(phenyl{[4-(piperidin-1-ylmethyl)phenyl]amino}methylidene)- 2,3-dihydro-1h-indole-6-carboxamide	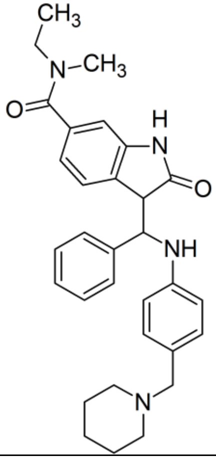
1B6C	2.60	0.249	0.269	---	---
1IAS	2.90	0.255	0.284	---	---
3KFD[69]	2.99	0.220	0.268	---	---

Table S2. Similarity values of each selected compound based on the crystal ligand

**PDB**	**Ligand**	**Resolution (Å)**	**Cpd-16 (pIC_{50} = 7.92)**	**Cpd-15 (pIC_{50} = 7.82)**	**Cpd-33 (pIC_{50} = 7.80)**
1RW8	580	2.40	0.51	0.52	0.47
1PY5	PY1	2.30	0.42	0.41	0.57
1VJY	460	2.00	0.40	0.41	0.44
2WOU	ZZF	2.30	0.33	0.34	0.35
2X7O	ZOP	3.70	0.49	0.48	0.34
3FAA	55F	3.35	0.30	0.33	0.46
3KCF	JZO	2.80	0.55	0.59	0.38
3HMM	855	1.70	0.31	0.32	0.78
3GXL	QIG	1.80	0.37	0.38	0.64
2WOT	ZZG	1.85	0.40	0.44	0.48

Table S3. Physicochemical properties of some ALK-5 ligands* and each crystallographic ligand

PDB	Ligand	nAtom	nC	nN	nO	CrippenLog	HybRatio	TopoPSA	MW	nHBAcc	nHBDon
-	15	52	23	5	1	3.905	0.217	62.97	385.180	5	0
-	16	57	25	5	2	3.676	0.280	72.20	427.201	6	0
-	33	39	18	5	0	5.921	0.111	91.83	333.105	5	1
1PY5	PY1	33	17	4	0	4.112	0.00	54.46	272.106	4	1
1RW8	580	38	18	3	0	4.348	0.222	30.71	293.133	3	0
1VJY	460	37	17	5	0	3.008	0.118	62.73	289.133	5	2
2WOU	ZZF	44	18	4	3	4.845	0.111	115.58	370.11	6	2
2X7O	ZOP	71	31	4	2	6.450	0.290	64.68	494.268	6	2
3FAA	55F	42	19	6	1	2.207	0.118	91.63	362.129	7	2
3KCF	JZO	47	21	4	2	2.971	0.190	58.56	360.159	6	0
3HMM	855	39	19	5	0	5.777	0.053	63.59	313.133	5	1
3GXL	QIG	45	21	6	0	3.354	0.143	61.14	354.159	6	2
2WOT	ZZG	60	26	4	4	7.171	0.192	87.62	458.195	4	1

*Araujo, S. C., Maltarollo, V. G., & Honorio, K. M. (2013). Computational studies of TGF-βRI (ALK-5) inhibitors: Analysis of the binding interactions between ligand–receptor using 2D and 3D techniques. European Journal of Pharmaceutical Sciences, 49(4), 542-549.

Table S4. Compounds selected from visual inspection regarding the main molecular interactions between some residues in the binding site and the studied ligands

Cpd	Structure	Cpd	Structure
1	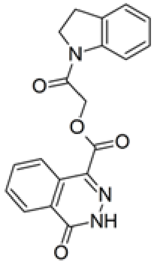	2	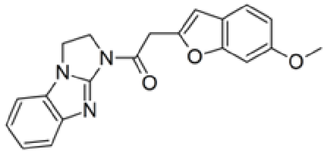
3	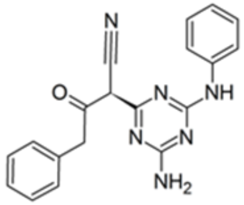	4	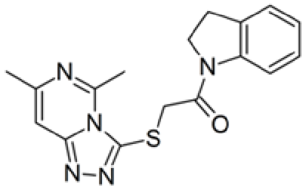
5	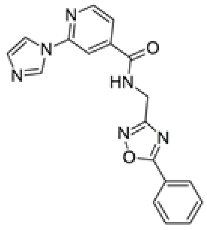	6	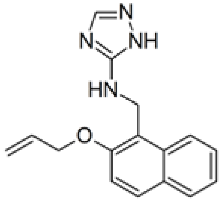

7

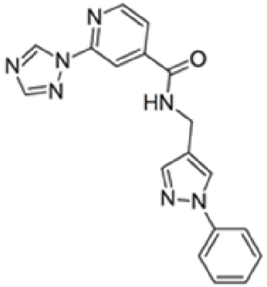

8

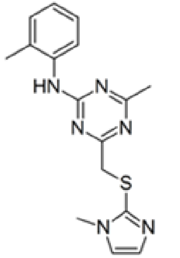

9

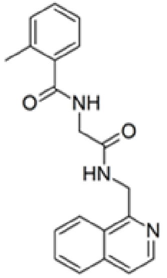

10

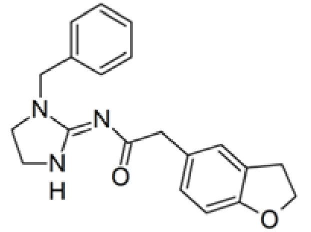

11

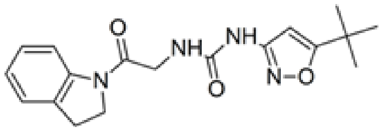

12

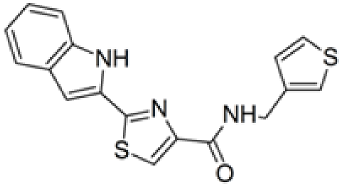

13

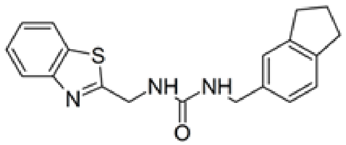

14

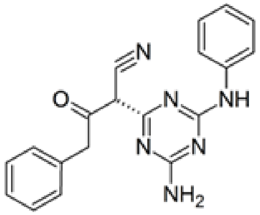

15 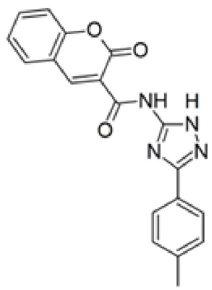

16 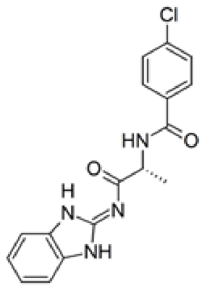

17 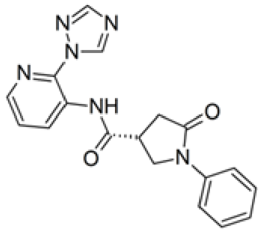

Table S5. Prediction of absorption (A), distribution (D), metabolism (M) and excretion (E) for the selected compounds and the crystal ligand

Property	Model Name	Range		Predicted Value					
		Bad	Good	Crystal	1	2	3	4	5
A	Water solubility (log mol/L)	>1	>-6	-2.873	-4.206	-3.281	-4.085	-3.154	-2.896
A	Caco2 permeability (log Papp in 10^{-6} cm/s)	<0.90	>0.90	1.145	1.229	0.532	0.81	1.445	1.28
A	Intestinal absorption (human) (% Absorbed)	<30%	>30%	100	99.017	93.351	91.456	96.341	97.441
A	Skin Permeability (log Kp)	>-2.5	<-2.5	-2.735	-2.74	-2.735	-2.755	-2.795	-2.735
A	P-glycoprotein substrate	No	Yes	No	Yes	No	Yes	No	Yes
A	P-glycoprotein I inhibitor	No	Yes	Yes	Yes	Yes	Yes	No	Yes
A	P-glycoprotein II inhibitor	No	Yes	Yes	Yes	Yes	Yes	No	No
D	VDss (human) (log L/kg)	<-0.15	>0.45	0.785	0.616	0.201	-0.557	-0.267	0.041
D	Fraction unbound (human) (FU)	>0	0	0.247	0	0.066	0	0.087	0.201
D	BBB permeability (log BB)	>0.3	<-1	-0.939	-0.259	0.424	-0.21	-0.684	-1.396
D	CNS permeability (log PS)	< -3	> -2	-2.996	-2.748	-2.079	-2.739	-2.634	-2.79
M	CYP2D6 substrate	not inhibit	inhibit	No	No	No	No	No	No
M	CYP3A4 substrate			Yes	Yes	Yes	Yes	Yes	Yes
M	CYP1A2 inhibitor			No	Yes	Yes	Yes	No	Yes
M	CYP2C19 inhibitor			Yes	Yes	Yes	No	No	Yes
M	CYP2C9 inhibitor			Yes	No	Yes	Yes	No	No
M	CYP2D6 inhibitor			No	No	No	No	No	No
M	CYP3A4 inhibitor			Yes	No	Yes	Yes	No	Yes
E	Total Clearance (log ml/min/kg)	<= -0.38	>-0.38	-0.104	0.468	1.144	-0.116	-0.016	0.478
E	Renal OCT2 substrate	No	Yes	No	Yes	Yes	No	No	Yes

Table S6. Prediction of toxicity for the new proposed compounds and the crystal ligand

Model Name	Range			Predicted Value				
	Bad	Good	Crystal	1	2	3	4	5
AMES toxicity	Yes	No	No	Yes	Yes	No	No	Yes
Max. tolerated dose (human) (log mg/kg/day)	>0.477	<=0.477	0.211	0.302	0.391	-0.045	-0.033	0.653
hERG I inhibitor	Yes	No	No	No	Yes	No	No	No
hERGII inhibitor	Yes	No	Yes	No	Yes	Yes	No	Yes
Oral Rat Acute Toxicity (LD 50) (mol/kg)			2.749	2.107	2.668	2.692	2.451	2.549
Oral Rat Chronic Toxicity (LOAEL) (log mg/kg_bw/day)			0.9	2.368	1.169	1.774	1.029	0.572
Hepatotoxicity	Yes	No	Yes	Yes	No	Yes	Yes	Yes
Skin Sensitisation	Yes	No	No	No	No	No	No	No
T. Pyriforms toxicity (log ug/L)	> 0.5	< 0.5	0.285	0.302	0.285	0.313	0.34	0.285
Minnow toxicity (log mM)	< - 0.3	> - 0.3	2.273	0.918	-1.183	-0.256	-1.298	2.294
